# L‐carnitine improves metabolic disorders and regulates apelin and apelin receptor genes expression in adipose tissue in diabetic rats

**DOI:** 10.14814/phy2.14641

**Published:** 2020-12-05

**Authors:** Neda Ranjbar Kohan, Mohammad Reza Tabandeh, Saeed Nazifi, Zahra Soleimani

**Affiliations:** ^1^ Department of Clinical Studies School of Veterinary Medicine Shiraz University Shiraz Iran; ^2^ Department of Basic Sciences Biochemistry Section Faculty of Veterinary Medicine Shahid Chamran University of Ahvaz Ahvaz Iran; ^3^ Department of Basic Sciences Histology Section Faculty of Veterinary Medicine Shahid Chamran University of Ahvaz Ahvaz Iran

**Keywords:** adipose tissue, apelin, APJ, L‐carnitine

## Abstract

Apelin is a new adipocytokine that acts as an endogenous hormone in various tissues through its receptor (APJ). This study aimed to investigate the effects of oral administration of L‐carnitine (LC) on the expression of Apelin and APJ in adipose tissue of experimentally induced insulin‐resistant and type 2 diabetic rats. In this experimental study, 60 male rats fed with high fat/high carbohydrate (HF/HC) diet. After 50 mg/kg intraperitoneally injection of streptozotocin (STZ) and confirmation of diabetes (FBS higher than 126 mg/dl), the animals were daily treated with 300 mg/kg LC for 28 days. At days 7, 14, and 28 of posttreatment, the expression of apelin and APJ in adipose tissue were determined using qPCR in diabetic, diabetic + LC treated, control, and control + LC treated groups. Apelin, insulin, TNF‐α, and IL1‐β were measured by the ELISA method. Results demonstrated that the rats fed with the HF/HC diet for 5 weeks were hyperinsulinemic and normoglycemic, while after STZ injection, they showed hyperinsulinemia and hyperglycemia with higher levels of HOMA‐IR. Apelin serum level, APJ and apelin gene expression in adipose tissue increased significantly with the development of diabetes compared to the control group. Treatment with LC for 14 days caused a reduction in apelin and APJ expressions in adipose tissue of diabetic rats. TNF‐α and IL1‐β levels were reduced in diabetic rats 14 days after their treatment with LC. The study results show that L‐carnitine could act as a new regulator in apelin gene expression in adipose tissue, improving the metabolic disorders in diabetic patients.

## Graphical abstract

The study investigated the effects of oral administration of L‐carnitine (LC) on the expression of Apelin and APJ in adipose tissue of experimentally induced insulin‐resistant and type 2 diabetic rats. In summary, the treatment with LC for 14 days caused a reduction in apelin and APJ expressions in adipose tissue of diabetic rats. Moreover, a decrease in the TNF‐α and IL1‐β levels could be observed. The results indicate that L‐carnitine could act as a new regulator in apelin gene expression in adipose tissue, improving the metabolic disorders in diabetic patients.

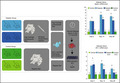

## 1 INTRODUCTION

Type 2 diabetes is a metabolic syndrome that is characterized by obesity and insulin resistance. Obesity has drawn increasing attention in the industrialized countries, and the number of obese and overweight individuals is increasing in alarming proportions worldwide. Some sites are metabolically active, like white adipose tissue (AT), liver, and immune cells. In obesity, the inflammatory process is activated in these metabolically active sites by an increase in the white AT mass (Kang et al., 2016). AT is an endocrine organ that produces and secretes many molecules that are generally called adipokines. This organ can interface with other central and peripheral organs through these adipokines. Secreted adipokines from AT contribute to the induction of insulin resistance in Diabetes mellitus type 2 (T2DM; El husseny et al., 2017). Some of the serum levels of adipokine can impact specific metabolic situations and affect the metabolic system. For example, an imbalance in adipokine secretion is observed in type 2 diabetes, hypertension, and cardiovascular disease. Obese individuals and the ones who have metabolic syndrome are known for an imbalance in their adipokine profiles. In this situation, decreased insulin sensitivity and other changes can be observed in the biochemical metabolites. Stress and visceral obesity development are strongly linked (Karalis et al., 2009).

Different studies have reported that AT‐derived hormones have an essential role in the complications associated with obesity and type 2 diabetes. Apelin is a newly identified adipokine that is produced and secreted by mature adipocytes and stimulating glucose uptake (Bertrand et al., 2015). Apelin is a bioactive peptide that derives from a 77‐amino‐acid precursor. It is an endogenous ligand that binds to a G‐protein‐coupled receptor called APJ (Lv et al., 2017). Recent findings have shown that human and mouse mature adipocytes produce and secrete apelin and APJ (Boucher et al., 2005). There is a relationship between obesity and the plasma levels of apelin; apelin upregulation and an increase in its plasma levels were reported in obesity in association with hyperinsulinemia. Boucher et al. (2005) reported increased plasma levels of apelin in obese individuals compared with control lean individuals. Many reports have demonstrated that apelin plays a physiological role in the regulation of glucose homeostasis and obesity. Yue et al. (2010) reported that apelin deficiency caused decreased insulin sensitivity in mice which had been fed with a high‐fat diet (HFD).

Dray et al. (2008) illustrated that the intravenous injection of apelin could cause increased glucose utilization in AT of insulin‐resistant mice. Heinonen et al. (2005) showed that there was a tight correlation between adipocyte‐secreted apelin and insulin. In vivo and in vitro studies have shown that insulin can directly affect the regulation of apelin gene expression in adipocytes. It was reported that protein kinase B phosphorylation, activated by insulin, in AT of mice fed with a HFD for 14 weeks was more sensitive compared to that which existed in the liver or muscle. Apelin production and its expression regulation in adipocytes or AT is strongly upregulated by insulin and tumor necrosis factor (TNF‐a; Castan‐Laurell et al., 2008).

Carnitine (β‐hydroxy‐γ‐N‐trimethylaminobutyric acid) is widely distributed in food from animal sources, but its availability in plants is limited. This term is derived from the Latin word “carnus.” Carnitine affects the metabolism of carbohydrate. Several complications, such as diabetes mellitus, hemodialysis, trauma, malnutrition, cardiomyopathy, obesity, fasting, drug interactions, endocrine imbalances, and other disorders, are supposed to be implicated in aberrations in carnitine regulation (Bene et al., 2018).

Studies have indicated that carnitine supplementation may be beneficial in fighting obesity. In obese rats that revealed insulin resistance, carnitine supplementation could ameliorate glucose tolerance and increase total energy utilization. In order to treat obesity, there is a rate‐limiting step, Carnitine palmitoyltransferase (CPT)‐1, in the fatty acid oxidation pathway. The modulation of CPT‐1 may affect energy metabolism and food intake (Bene et al., 2018). The mechanisms of immune compromise in type 2 diabetes are unknown. It can be due to oxidative damage and mitochondrial dysfunction. This hypothesis was tested using mitochondrial targeting nutrients in a diabetic rat model. Treatment with a combination of mitochondrial targeting nutrients, such as carnitine, revealed that it might be effective in ameliorating the immune function in type 2 diabetes by increasing the mitochondrial function, decreasing oxidative damage, and delaying cell death in the immune organs and blood (Hao et al., 2009). In another study, carnitine supplementation improved glucose tolerance and increased energy expenditure in obese mice with determined insulin resistance. Patients with type 2 diabetes were found to be at increased risk of carnitine deficiency (Rogero & Calder, 2018).

In this study, the experimental model of feeding mice with high‐fat/carbohydrate diet was followed. The study was aimed to investigate the effects of diabetes induction on apelin and APJ gene expression in AT of a well‐established animal model of type 2 diabetes and evaluate the effects of L‐carnitine on AT expression of apelin and APJ gene.

## 2 MATERIALS AND METHODS

### 2.1 Animals

In this study, 60 male Wistar rats with an average weight of (200 ± 12 g) and age of (2–3 months) were selected from the center of laboratory animals of the Faculty of Veterinary Medicine of Shahid Chamran University, Ahvaz, Iran. A temperature‐controlled room was prepared for the rats (at 23 ± 1°C) with 12‐hr light/dark cycles, and there was free access to rat chow (Pars, Iran) and water ad libitum. Before starting the experiment, the rats experienced acclimatization for 7 days. This experiment was performed under the approval of the State Committee on Animal Ethics, Shiraz University, Shiraz, Iran. The recommendations of the European Council Directive (86/609/EC) of November 24, 1986, regarding the protection of animals used for experimental purposes, were also followed.

### 2.2 Sampling

Serum and tissues were taken on days 0, 14, and 28 after the induction of diabetes and treatment with LC. The animals were euthanized with a combination of 100 mg/kg of ketamine and 10 mg/kg of xylazine. Right after the euthanasia, blood samples were collected, and sera were separated and then stored at −20°C until further use. Visceral AT was separated and kept at −70°C until further use. Measuring the absolute body weight of each rat from each group was performed at the end of the HF/HC feeding and LC treatment period.

### 2.3 Experimental design

The rats (*n* = 60) were randomly divided into four equal groups (*n* = 15). Two groups were fed with high‐energy diet [prepared by adding 20% sucrose (w/w) and 10% beef tallow (w/w) into diets] (Nazari et al., 2016) for 5 weeks and called High fat/High carbohydrate (HF/HC; *n* = 30); then, they received a single dose of streptozotocin (30 mg/kg, i.p; STZ, Sigma, Germany; Qian et al., 2015). Five days after STZ treatment, glucose was measured by hand‐held glucometer (EasyGluco, South Korea) in HF/HC. The elevation of serum glucose above 7.5 mmol/L was considered as an index and confirmation of diabetes induction (Nazari et al., 2016). One diabetic group (*n* = 15) received LC 300 mg kg^−1^ day^−1^ (Bazotte & Bertolini, 2012) in drinking water concomitant with HF/HC diets for 28 days. The other diabetic group (*n* = 15; diabetic control) received only the HF/HC diet for 28 days. The other two groups consumed regular diets for 5 weeks and served as control groups (*n* = 30). One control group (*n* = 15) received normal diet while the other control group (*n* = 15) was treated with 300 mg kg^−1^ day^−1^ LC (*n* = 15) in drinking water for the same period.

### 2.4 Plasma biochemistry assay

Cholesterol, high‐density lipoprotein cholesterol (HDL‐C), low‐density lipoprotein cholesterol (LDL‐C), the plasma glucose, and triglyceride (TG) levels were measured using commercially available kits (Pishtazteb, Iran). The serum levels of Apelin (East Biopharm, Mainland, China) and insulin (KOMA BIOTECH INC, South Korea) were determined by rat‐specific ELISA kits using a multiplate ELISA reader (BioTek, CA, USA). The assay sensitivity for insulin was 0.75 μIU/ml. TNF‐α and IL‐1β levels in the serum were determined using ELISA kits explicitly designed for rats (KOMA BIOTECH INC, South Korea). The assay sensitivities for TNF‐α and IL‐1β were 45pg/ml and 15 pg/ml, respectively.

### 2.5 Homeostasis model assessment of basal insulin resistance estimation

For the homeostasis model assessment of basal insulin resistance (HOMA‐IR), the following equation was used: HOMA‐IR = Fasting insulin level (μU/ml) × fasting blood glucose (mmol/L) divided by 22.5 (23). Lower HOMA‐IR values indicated greater insulin sensitivity, whereas higher HOMA‐IR values indicated lower insulin sensitivity (i.e. insulin resistance; Fan et al., 2014).

### 2.6 Isolation of RNA

Total RNA was isolated from 100 mg of AT using RNX TM isolation reagent according to the manufacturer's procedure (Cina Clon, Iran). Possible DNA contamination was removed by the treatment of RNA (1 μg) with DNase I (2 U/μl) for 1 hr at 37°C (Vivantis, Malaysia). The concentration of extracted RNA was calculated at the wavelength of 260 nm using NanoDrop spectrophotometer (Eppendorf, Germany). For detecting the purity of the RNA, the optical density (OD) ratio at 260/280 nm was determined, and samples with a ratio >1.8 were used for cDNA synthesis. Reverse transcription was carried out using the Rocket Script RT PreMix kit with 1 μg of RNA and random hexamer primers based on the manufacturer's protocol (Bioneer Corporation, South Korea). Reverse transcription was carried out at 42°C for 90 min, followed by incubation at 70°C for 5 min. cDNAs were stored at −20°C to be used later for real‐time polymerase chain reaction (PCR).

### 2.7 Real‐time polymerase chain reaction analysis

To evaluate the expression levels of *APJ* and *Apelin* in AT, real‐time PCR analysis (Light‐Cycler 480; Roche, Germany) was performed. The relative expression levels of *APJ and apelin* transcripts were compared to rat *GAPDH* as the housekeeping gene.

Specific sets of primers (Bioneer, South Korea) designed for this study were as follows: *Apelin* (GenBank accession NO: NM_031612.3): F: 5′‐TGGAAGGGAGTACAGGGATG‐3′, R: 5′‐TCCTTATGCCCACT‐3′; *Apj* (GenBank accession NO: NM_031349.2): F: 5′‐GGACTCCGAATTCCCTTCTC‐3′, R: 5′‐CTTGTGCAAGGTCAACCTCA‐3′; *Gapdh* (GenBank accession NO: NM_NM‐001034055): F: 5′‐CTCATCTACCTCTCCATCGTCTG‐3′, R: 5′‐CCTGCTCTTGTCTGCCGGTGCTTG‐3′.

Reaction volume for the analysis of *Apelin* and *APJ* gene expressions was performed using 12.5 μl, containing 6.25 μlq PCRTM Green Master Kit for SYBR Green I® (Jena Bioscience, Germany), 0.25 μl of each primer (200 nM), 3 μlcDNA (~100 ng), and 2.25 μl nuclease‐free water. The PCR protocol consisted of a 5‐min denaturation at 95°C, followed by 45 cycles at 95°C for 15 s and 60°C for 30 s. Reactions were performed in triplicate.

All runs included one negative‐template control consisting of PCR‐grade water instead of cDNA. Relative quantification was performed according to the comparative 2^−ΔΔCt^ method and using Lightcycler 96® software. The validation of the assay, that is, checking that the primer used for the target genes and internal reference genes had similar amplification efficiencies, was performed as described previously. All qPCR analysis was performed according to the Minimum Information for Publication of Quantitative Real‐Time PCR Experiments (MIQE) guideline (Bustin et al., 2009).

### 2.8 Statistical analysis

The IBM SPSS 18 software was used for all statistical analyses. Descriptive statistics were presented as means ± *SE*. Means of each variable in the treatment groups and at various time points were compared by using a two‐way analysis of variance. Group, time, and their interaction term were considered as fixed effects in the model. In significant cases, an adjusted comparison of means was undertaken using the Sidak post hoc test. In the case of high variability among data and a nonhomogenous variance, the data were transformed. In most cases, the variance became homogenous after applying a logarithmic transformation. However, the daily animal weight comparison among each experimental group was performed using nonparametric analysis of variance (Kruskal–Wallis test) followed by the Mann–Whitney *U* test. In all performed analyses, values *p* < .05 were considered statistically significant.

## 3 RESULTS

### 3.1 Effects of L‐carnitine supplementation on insulin resistance markers

At the end of the 5‐week feeding with HF/HC, no significant changes were observed in the serum glucose level while its level significantly increased after STZ treatment (Table 1).

**Table 1:** Effects of LC on glucose, insulin, and HOMA‐IR levels in diabetic rats fed with an HF/HC diet

	Day 0	Day 14	Day 28
Glucose (mg/dl)			
Control	98/28 ± 5/60^a^	101/28 ± 4/47^c^	94/82 ± 6/99^c^
Diabetic	109/68 ± 7/41^a^	184/88 ± 13/92^a^	207/70 ± 15/69^a^
Diabetic + L‐carnitine	106/30 ± 4/51^a^	136/08 ± 7/14^b^	155/48 ± 14/80^b^
Control + L‐carnitine	93/17 ± 2/14^a^	93/10 ± 7/06^c^	94/16 ± 4/39^c^
Insulin (pmol/L)			
Control	93/94 ± 5/38^b^	95/88 ± 4/85^c^	96/44 ± 6/15^c^
Diabetic	181/70 ± 15/00^a^	188/70 ± 8/70^a^	173/20 ± 14/70^a^
Diabetic + L‐carnitine	172/06 ± 11/85^a^	126/86 ± 9/92^b^	112/98 ± 6/71^b^
Control + L‐carnitine	92/22 ± 4/60^b^	94/76 ± 2/82^c^	95/10 ± 5/91^c^
HOMA‐IR control			
Control	3/30 ± 0/35^b^	3/46 ± 0/29^c^	3/24 ± 0/31^c^
Diabetic	7/18 ± 1/06^a^	12/46 ± 1/48^a^	12/65 ± 1/26^a^
Diabetic + L‐carnitine	6/50 ± 0/55^a^	6/13 ± 0/62^b^	6/15 ± 0/50^b^
Control + L‐carnitine	3/04 ± 0/14^b^	3/13 ± 0/25^c^	3/18 ± 0/25^c^

Note

Data are expressed as means ± *SE*. Different letters (a, b, and c) demonstrate significant differences between groups on each day at *p* < .05.

Abbreviations: HF/HC, High fat/high carbohydrate; HOMA‐IR, Homeostatic model assessment of insulin resistance; LC, L‐carnitine.

In the diabetic group, compared to the control group, higher levels of insulin and HOMA‐IR were observed after diabetes induction (*p* < .05, Table 1). These results showed that HF/HC diet feeding and STZ treatment could lead to apparent insulin resistance, accompanied by elevated fasting blood glucose, insulin, and HOMA‐IR index, compared to the control group. Hyperglycemia and hyperinsulinemia were reduced, and HOMA‐IR was elevated in diabetic rats fed with HF/HC and treated with LC supplementation for 14 and, in particular, 28 days (Table 1).

### 3.2 Effects of L‐carnitine supplementation on body weight change

Regarding the body weight, a significant difference was seen in the groups fed with HF/HC and a regular diet. Compared to the control group, the elevation of body weight, in a time‐dependent manner, could be attributed to the feeding with a high‐calorie diet for 5 weeks (*p* < .05, Table 2). No significant changes were observed in the body weight of the diabetic rats, which were treated with LC for 14 or 28 days (*p* > .05, Table 2). Also, no significant change was seen in the body weight of healthy rats after 28 days.

**Table 2:** Effects of LC on body weight in diabetic rats fed with an HF/HC diet. Rats were fed with regular chow diet (control) or HF/HC diet for 5 weeks (diabetic)

	Day 0	Day 14	Day 28
Weight (g)			
Control	217 ± 8/9^b^	248/2 ± 16/52^b^	256 ± 9/35^b^
Diabetic	277 ± 25/37^a^	311/4 ± 20/88^a^	347 ± 23/78^a^
Diabetic + L‐carnitine	275 ± 32/21^a^	256/2 ± 23/25^b^	272/2 ± 28/17^b^
Control + L‐carnitine	219 ± 6/05^b^	242/2 ± 7/12^b^	248/6 ± 11/54^b^

Note

Data are presented as means ± *SE*. Different letters (a, b, and c) demonstrate significant differences among groups on each day at *p* < .05.

Abbreviations: HF/HC, High fat/high carbohydrate; LC, L‐carnitine.

### 3.3 Effects of L‐carnitine supplementation on alteration of serum levels of apelin

**Figure 1:**
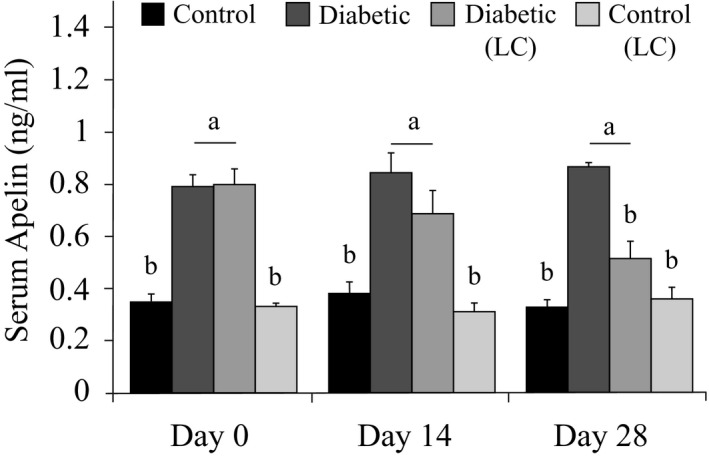
Effects of LC on the serum levels of apelin in the diabetic rats fed with an HF/HC diet. Rats were fed with regular chow diet (control) or HF/HC diet for 5 weeks (diabetic). Rats fed with HF/HC were treated with LC (300 mg kg^−1^ day^−1^) from the first day of diabetes confirmation to 14 or 28 days after that (Diabetic + LC,*n* = 5/ group). Data are expressed as means ± *SE*. Different letters (a, b) demonstrate significant differences among groups on each day at*p* < .05. LC, L‐carnitine; HF/HC, High fat/high carbohydrate

The diabetic group showed significantly higher levels of serum apelin on all days of the experiment compared to the control group (Figure 1). The administration of LC to diabetic rats for 14 days caused no significant changes in the serum levels of apelin while LC administration for 28 days led to a significant decrease in the serum levels of apelin (*p* < .05). LC administration for 14 or 28 days had no significant effects on the serum levels of apelin in the healthy rats. The data in Figure 1 are expressed as means ± *SE*, and the letters (a and b) demonstrate significant differences among groups on each day at *p* < .05.

### 3.4 Effects of L‐carnitine on the expression of *apelin*and *APJ* in AT of diabetic rats

**Figure 2:**
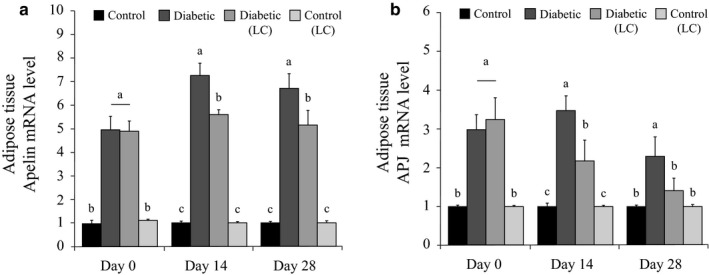
Effects of LC on the expression of*apelin*and*APJ*mRNA (on days 0, 14, and 28 of the experiment) in adipose tissue of the diabetic rats fed with an HF/HC diet. Rats were fed with regular chow diet (control) or HF/HC diet for 5 weeks (diabetic). Rats fed with HF/HC were treated with LC (300 mg kg^−1^ day^−1^) from the first day of diabetes confirmation to 14 or 28 days after that (Diabetic + LC,*n* = 5/group). (a)*Apelin*mRNA level, (b)*APJ*mRNA level. Data are expressed as means ± *SE*. Different letters (a, b, and c) demonstrate significant differences among groups on each day at*p* < .05. LC, L‐carnitine; HF/HC, High fat/high carbohydrate

In diabetic rats, compared to the healthy ones, a significant increase was observed in the expression level of apelin AT on 14 and 28 days after diabetes induction (*p* < .05, Figure 2a). The expression of apelin in the AT of diabetic rats decreased significantly after LC treatment for 14 and 28 days (*p* < .05, Figure 2a).

APJ AT expression showed upregulation in the diabetic rats compared to the control rats after 14 and 28 days of diabetes induction. Treatment with LC for 14 and 28 days caused a significant reduction in the gene expression of diabetic rats (*p* < .05, Figure 2b).

In general, the results demonstrated the effects of LC on downregulation of AT apelin and APJ expressions in diabetic rats. LC‐treated control rats did not show any significant changes in terms of these genes' expressions in their AT (*p* > .05, Figure 2a,b). The data presented in Figure 2 are expressed as means ± *SE*, and the different letters (a, b, and c) demonstrate significant differences among groups on each day at *p* < .05.

### 3.5 Effects of L‐carnitine supplementation on serum lipids

In Table 3, the lipid profile, which includes LDL, HDL, cholesterol, and TG levels in diabetic rats after their treatment with LC, is shown on all days of the experiment.

**Table 3:** Effects of LC on the levels of serum lipids in the diabetic rats fed with an HF/HC diet. Rats were fed with regular chow diet (control) or HF/HC diet for 5 weeks (diabetic)

	Day 0	Day 14	Day 28
Triglycerides (mg/dl)			
Control	93/92 ± 5/22^a^	92/02 ± 4/54^a^	92/92 ± 4/04^a^
Diabetic	145/86 ± 8/41^b^	211/96 ± 9/79^c^	267/48 ± 14/36^d^
Diabetic + L‐carnitine	141/96 ± 7/81^b^	158/86 ± 11/99^b^	148/36 ± 10/72^b^
Control + L‐carnitine	92/9 ± 4/74^a^	90/34 ± 3/79^a^	91/09 ± 2/24^a^
Cholesterol (mg/dl)			
Control	86/16 ± 3/44^b^	90 ± 4/13^b^	92/66 ± 3/92^b^
Diabetic	104/06 ± 5/4^ab^	151/5 ± 7/21^a^	227/34 ± 10/23^a^
Diabetic + L‐carnitine	111/98 ± 4/23^a^	131/04 ± 8/67^a^	114 ± 8/81^b^
Control + L‐carnitine	89/63 ± 5/39^ab^	88/5 ± 5/21^b^	89/74 ± 3/87^b^
LDL‐C (mg/dl)			
Control	95/58 ± 5/06^a^	93/16 ± 5/99^a^	95/86 ± 4/75^a^
Diabetic	133/46 ± 8/73^b^	181/8 ± 17/14^c^	265/42 ± 20/89^d^
Diabetic + L‐carnitine	129/92 ± 5/57^b^	128/66 ± 5/20^b^	155/7 ± 10/72^b^
Control + L‐carnitine	94/10 ± 6/43^a^	93/1 ± 6/55^a^	93/68 ± 5/49^a^
HDL‐C (mg/dl)			
Control	43/88 ± 3/3^a^	44/26 ± 2/51^a^	45/34 ± 4/64^a^
Diabetic	34 ± 2/01^a^	33/76 ± 2/20^b^	29/38 ± 2/38^b^
Diabetic + L‐carnitine	35/12 ± 1/97^a^	37/86 ± 1/41^a^	38/1 ± 2/34^a^
Control + L‐carnitine	42/94 ± 1/72^a^	41/34 ± 3/69^a^	41/28 ± 2/23^a^

Note

Data are expressed as means ± *SE*. Different letters (a, b, and c) demonstrate significant differences among groups on each day at *p* < .05.

Abbreviations: HDL‐C, High‐density lipoprotein cholesterol; HF/HC, High fat/high carbohydrate; LC, L‐carnitine; LDL‐C, Low‐density lipoprotein cholesterol.

The results showed a significant increase in the TG and LDL serum levels in the diabetic rats compared to healthy rats at the end of the HF/HC feeding period (*p* < .05). Also, there was no significant increase in the cholesterol and HDL levels in the HF/HC‐fed rats compared to the healthy rats. However, 14 and 28 days after the induction of diabetes, an elevation was observed in the LDL, cholesterol, and TG serum levels while a significant reduction was observed in the HDL serum level of the diabetic rats compared to the healthy ones (*p* < .05, Table 3).

A significant decrease was observed in the TG and LDL serum levels of rats treated with LC and fed with HF/HC for 14 and 28 days; however, the HDL serum level increased only 28 days after LC treatment compared to the untreated diabetic rats (*p* < .05, Table 3).

### 3.6 Effects of L‐carnitine supplementation on serum tumor necrosis factor‐α and interleukin‐1β in diabetic rats

To evaluate the effects of L‐carnitine supplementation on the inflammation following an HF/HC diet feeding, the serum levels of the TNF‐α and interleukin‐1β (IL‐1β), were measured in the treated and nontreated animals.

**Figure 3:**
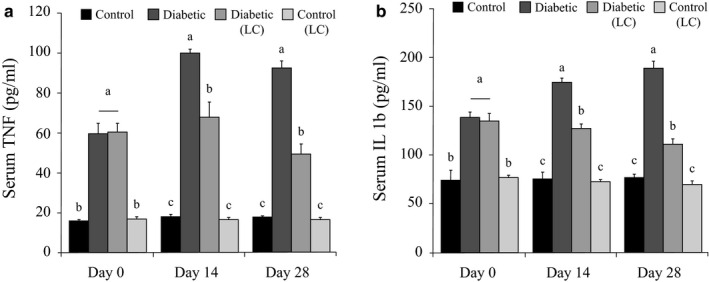
Effects of LC on the serum levels of TNF‐α and IL‐1β in the induced diabetic rats fed with an HF/HC diet. Rats were fed with regular chow diet (control) or HF/HC diet for 5 weeks (diabetic). HF/HC fed rats were treated with LC (300 mg kg^−1^ day^−1^) from the first day of diabetes confirmation to 14 or 28 days after that (Diabetic + LC,*n* = 5/group). (a) Serum TNF‐α, B. Serum IL‐1β. Data are expressed as means ± *SE*. Different letters (a, b, and c) demonstrate significant differences between groups in each day at*p* < .05. LC, L‐carnitine; HF/HC, High fat/high carbohydrate; TNF‐α, Tumor necrosis factor‐α; IL‐1β, interleukin‐1β

According to Figure 3a,b, which show changes in the serum levels of TNFα and IL‐1β, the serum levels of TNFα and IL‐1β of the diabetic rats were significantly higher than those of the healthy control rats at all sampling days (*p* > .05). Feeding the diabetic rats with an HF/HC diet and treating them with LC significantly reduced the serum levels of TNFα and IL‐1β in them on days 14 and 28 compared to the untreated diabetic rats (*p* < .05, Figure 3a,b). LC treatment had no significant effects on the serum levels of TNFα and IL‐1β in the healthy rats (*p* > .05). The data presented in Figure 3 are expressed as means ± *SE*, and the different letters (a, b, and c) demonstrate significant differences among groups on each day at *p* < .05.

## 4 DISCUSSION

The AT is associated with central and peripheral organs by the synthesis and secretion of molecules known as adipokines. The levels of some adipokines are associated with body‐specific metabolic conditions and can directly affect the metabolic homeostasis. Some disorders have been observed in the adipokine regulation in obesity, type 2 diabetes, and cardiovascular disease. Obese people are exposed to different metabolic disorders, such as type 2 diabetes, lipid disorders, hypertension, and cardiovascular disease (Lu et al., 2014; Mandviwala et al., 2016). The most important consequence of obesity is the development of insulin resistance. The detection of AT hormones or adipocytokines and changes in the protein levels and gene expressions of these hormones in different tissues, as well as the strong relationship between these alterations and insulin resistance in obese patients, have made some researchers attempt to identify the mechanism of metabolic disorders following obesity and find new treatments. Apelin is one of the most important of these adipokines. Apelin is a new hormone that has been identified recently and its role in molecular changes is associated with type 2 diabetes, obesity, and cardiovascular disorders (Kleinz et al., 2005). Recent studies have shown that there is a disorder in the regulation of the expression and secretion of apelin and its receptor (APJ) in the AT of obese and diabetic patients (Alfarano et al., 2015).

In this study, HF/HC‐fed rats were used for a reasonably long time as models of obesity and diabetes to study the effects of L‐carnitine on the apelin gene expression in AT. The results of this study showed that in HF/HC‐fed rats, pathophysiological disorders developed in manners similar to those in the metabolic syndrome. This condition increased body weight, blood cholesterol, triglyceride, LDL, and glucose, on one hand, and decreased HDL with hyperinsulinemia and insulin resistance, on the other hand, of the rats in the treated group compared with those in the control group. L‐carnitine leads to a decrease in the level of LDL, blood cholesterol, and glucose uptake. Ahmadi et al. (2016), found that L‐carnitine complements could affect the serum level of adipocytokines and increase the plasma levels of adiponectin and leptin. However, the effects of L‐carnitine on the function of the apelin system in the AT have remained unknown until now. Therefore, this study aimed to evaluate the protective effects of L‐carnitine on the apelin system in the AT. In this study, the rats fed with an HF/HC diet were selected as a model of obesity and diabetes.

The results of this research showed that L‐carnitine administration to diabetic rats for 14 or 28 days could decrease the hyperglycemia and hyperinsulinemia, on one hand, and increase the HOMA‐IR index, on the other hand. These changes were more clearly observable on day 28 of the administration of L‐carnitine supplementation.

Insulin resistance is created in specific tissues such as AT, muscle tissue, and liver. Insulin resistance in the liver results in a disorder in the glycogen synthesis and some defects in the production of glucose (Gao et al., 2010). In this study, feeding rats with a high‐calorie diet for 5 weeks resulted in an increase in body weight compared to the rats in the control group at all days of the experiment. After the induction of diabetes, the weight of diabetic rats was higher than the control group on days 14 and 28. Treatment with L‐carnitine for 14 or 28 days caused a significant decrease in the body weight of diabetic rats compared to the control. The cause of weight gain during this time can be attributed to excessive AT growth due to a negative energy balance. The expansion of fat deposits could have been due to an increase in the numbers of adipocytes (hyperplasia) and their size (hypertrophy). A negative energy balance leads to the spread of fat deposits that can, in turn, increase the inflammatory adipokines. By enhancing insulin resistance, body glucose consumption will reduce. Then, due to the diabetogenic properties of visceral AT and beta‐cell degradation, type 2 diabetes is developed, and hyperglycemia will appear (Frayn, 2002).

In line with the results of this study, Cave et al. (2008) showed that adding L‐carnitine complement to the diet of obese rats which had insulin resistance could ameliorate glucose tolerance and increase energy consumption. The possible mechanism through which L‐carnitine functions can be related to the enzyme CPT‐1, limits fatty acids oxidation and affects the energy metabolism and food intake (Rogero & Calder, 2018). These days, more and more studies are attending to obesity management, which is about the consequences of inhibiting and increasing the activity of this enzyme. Also, Aja et al. (2008), showed that brain CPT‐1 stimulation could lead to a reduction in food intake and cause weight loss. Other pieces of evidence also suggest that L‐carnitine supplements can be useful in obesity and diabetes. It was shown that in obese rats with insulin resistance, L‐carnitine supplements could improve glucose tolerance and increase energy consumption (Uysal et al., 2005).

The results of the lipid profile in our study indicated that the oral L‐carnitine supplementation could decrease the concentrations of TG, cholesterol, and LDL, on one hand, and increase (HDL) plasma level, on the other hand. Emami Naini et al. (2012), showed that oral L‐carnitine supplementation could lead to a decrease in the TG and an increase in the HDL plasma levels (Emami Naini et al., 2012). A possible explanation for these changes could be due to the increase in the activity of CPT and the respiratory chain enzymes, and L‐carnitine supplementation prompts an increased oxidative capacity in the liver and muscle. These effects can be caused by an increase in the unsaturated fatty acids in the mitochondrial phospholipids or an increase in the membrane fluidity (Le Borgne et al., 2017). Kennard and Singer (2017), reported that L‐carnitine supplementation decreased the concentrations of TG and cholesterol and increased the HDL plasma level in dialysis patients. However, it did not cause any significant changes in the LDL concentration.

During the last decades, inflammation has been recognized as a key factor in obesity and type 2 diabetes. Chronic inflammation caused by long‐term feeding with a high‐energy diet leads to increased production of acute‐phase proteins and inflammatory cytokines, such as TNF‐α and IL‐1β.

In this study, the serum TNF‐α and IL‐1β levels significantly increased in the diabetic rats compared to the rats in the control group in all experimental days. The prominent feature in rats fed with HF/HC was chronic inflammation. This circumstance could have happened due to the infiltration of macrophages into the adipose cells, leading to high production of acute‐phase proteins and inflammatory cytokines, like TNF‐α and IL‐1β. In Nakamura et al. (2014) study, the upregulation in the TNF‐α gene was seen in the AT and the serum of diabetic rats. At the same time, the downregulation of TNF‐α was observed in fat individuals who had undergone weight loss. However, many studies have shown that in obese AT, the mRNA levels of TNFα and its protein increase, and there is controversy about the relationship between obesity and TNFα concentration. A variety of experimental and clinical studies suggest that TNFα may act as an essential auto/paracrine regulator in fat cells which limits the AT spreading but increases insulin resistance, leading to some metabolic disorders (Than et al., 2015). There is a positive correlation between the mRNA expression level of TNF‐α and hyperinsulinemia in AT (Elochukwu et al., 2017). In this study, L‐carnitine treatment significantly reduced the serum TNFα and IL‐1β levels in HF/HC rats on all days of the experiment compared to the control rats.

It has been demonstrated that changes in the plasma apelin concentrations are significantly associated with changes in the plasma triglyceride, glucose, TNF‐α, and HOMA‐IR concentrations (Bertrand et al., 2015).

The results of Than et al. (2015) and Heinonen et al. (2005) showed a positive correlation between the apelin and TNFα expressions in AT. They observed a positive correlation between apelin plasma level and body mass index. Because elevated inflammatory cytokines in obesity can accelerate the expression and secretion of apelin, it is hypothesized that downregulation of apelin in obese diabetic rats treated with LC may be associated with the anti‐inflammatory action of LC.

Plasma apelin in obese animals and humans has a high concentration (Arica et al., 2018; Boucher et al., 2005). In 2005, Boucher et al. showed that apelin was synthesized and secreted in adipocytes and that there was a close relationship between apelin and insulin in in vivo and in vitro conditions (Boucher et al., 2005). Apelin expression in the AT of obese animal models increased along with hyperinsulinemia (Boucher et al., 2005).

In the present study, a significant increase was observed in the serum level of apelin in the HF/HC‐fed rats during all sampling days compared to the control group. Furthermore, the treatment of diabetic rats with L‐carnitine for 14 days did not have a significant effect on the serum apelin while diabetic rats that were treated with LC for 28 days showed a significantly reduced level of serum apelin. Also, the results indicated that the LC treatment could efficiently reduce the overexpression of apelin and APJ in AT caused by an HF/HC diet. These results showed that the serum level of apelin and the gene expressions of apelin and APJ were subject to similar changes. This finding suggests that in a diabetic state, simultaneous changes can occur in the gene expressions of apelin and APJ, which together can intensify the function of this hormone in the target tissue.

It has been proven that apelin is produced in adipocytes and that its concentration increases in obese individuals. Apelin can also increase glucose uptake by AMPK and the suppression of lipolysis (Yue et al., 2010). In fact, it can be stated that the increase in the plasma level of apelin is a compensatory mechanism activated during insulin resistance because it has been found to increase the glucose uptake in the skeletal muscle and AT and repress lipolysis in healthy and insulin‐resistant rats (Bertrand et al., 2015).

Miguel‐Carrasco et al. (Miguel‐Carrasco et al., 2008) showed that long‐term administration of L‐carnitine could reduce the severity of inflammation associated with metabolic disorders in rats. Due to an increase in the inflammatory cytokines in obesity that can accelerate the expression and secretion of apelin, it is hypothesized that any decrease in the gene expression of apelin in obese and diabetic rats treated with L‐carnitine can be related to the inflammatory effects of L‐carnitine. Therefore, it is expected that following the administration of L‐carnitine, the apelin gene expression will reduce and the inflammatory and metabolic disorders will attenuate. An increased apelin gene expression can be considered as a compensatory mechanism against the development of insulin resistance disorders in obese patients.

## 5 CONCLUSION

To summarize, the results of the present study showed that apelin and APJ (apelin receptor) gene expressions increased in AT of rats fed by HF/HC, and these changes were significantly associated with an increase in the serum apelin and insulin, body weight, insulin resistance, inflammatory markers, and atherogenic lipid profiles. The treatment of diabetic rats with L‐carnitine decreased the apelin gene expression, improved insulin sensitivity and inflammatory markers, and increased the body weight in the diabetic rats. These results indicated that L‐carnitine could act as a new regulator of apelin gene expression in AT and improve metabolic disorders in diabetic patients.

## CONFLICT OF INTEREST

The authors declare that they have no conflicts of interest.

## AUTHOR CONTRIBUTIONS

All authors contributed to all parts of the study from designing the study to writing and preparing the manuscript. Neda Ranjbar Kohan and Mohammad Reza Tabandeh contributed in study design, performing the study, sampling, data collection and analysis. Saeed Nazifi and Zahra Soleimani helped in study performing and preparing the manuscript. All authors read and approved the final manuscript.

## ETHICAL APPROVAL

The trial was approved by the members of the state commission on animal ethics, Shiraz University, Shiraz, Iran (IACUC no: 4687/63). Additionally, the advice of European Council Directive (86/609/EC) of November 24, 1986, concerning the protection of animals, was fully considered for the trial procedures.
